# Real-Time Prescription Benefit Tool Availability and Prescription Medication Fill Rates: A Post Hoc Analysis of a Cluster Randomized Clinical Trial

**DOI:** 10.1001/jamahealthforum.2026.2739

**Published:** 2026-08-14

**Authors:** Roger Ying, Prianca Padmanabhan, Adam Szerencsy, Ateev Mehrotra, Leora I. Horwitz, Sunita M. Desai

**Affiliations:** 1 Department of Population Health, NYU Grossman School of Medicine, New York, New York; 2 Department of Health Services, Policy, and Practice, Brown University School of Public Health, Providence, Rhode Island

## Abstract

**Importance:**

Patients frequently forgo filling prescriptions due to high out-of-pocket costs. Real-time prescription benefit (RTPB) tools that recommend available lower-cost, clinically equivalent medications to prescribing clinicians may increase the likelihood that patients fill their prescriptions.

**Objective:**

To determine whether availability of an RTPB tool increases prescription fill rates.

**Design and Setting:**

This post hoc analysis of a cluster randomized clinical trial included medical practices within an urban ambulatory clinical network randomized to the RTPB tool between January and December 2021. Outpatient prescriptions that were eligible for an RTPB recommendation during the study period were analyzed. Data analyses were performed from October 18, 2022, to August 9, 2024.

**Intervention:**

An electronic health record–integrated RTPB tool that displays available lower-cost and clinically equivalent alternatives to the initiated prescription at the point of prescribing.

**Main Outcome and Measure:**

The primary outcome measured whether a prescription was filled.

**Results:**

Of 1 386 577 outpatient prescriptions at randomized practices during the trial period, 38 289 (2.8%) were included in the analytic sample. Across all orders, the availability of the RTPB tool did not impact the proportion of orders filled (54% and 55% in the control and RTPB groups, respectively; adjusted difference: 1.2 percentage points [pp]; 95% CI, −1.3 to 3.7 pp). However, in the quartile of drug classes with the highest out-of-pocket costs (average out-of-pocket cost for a 30-day fill of >$120.83), fill rates increased from 33% in the control group to 49% in the RTPB group (adjusted difference: 14.5 pp; 95% CI, 8.4-20.6 pp). Similar increases were not detected in lower-cost drug classes. Increases in fill rates within the highest out-of-pocket cost drug classes were largest for patients in the lowest-income communities served by the health system (30.3 pp; 95% CI, 19.5-41.1 pp) but not substantial in the highest-income communities (1.0 pp; 95% CI, −10.2 to 12.2 pp).

**Conclusions and Relevance:**

In this post hoc analysis of a cluster randomized clinical trial, there was no change in overall prescription fill rates, but among high-cost drugs, the RTPB tool increased fill rates, particularly among patients from low-income communities. However, RTPB recommendations were made for a small proportion of orders, limiting the applicability of the findings to a narrow segment of the randomized population.

**Trial Registration:**

ClinicalTrials.gov Identifier: NCT04940988

## Key Points

**Question:**

Do real-time prescription benefit (RTPB) tools increase prescription fill rates?

**Findings:**

In this post hoc analysis of a cluster randomized clinical trial encompassing 38 289 eligible prescriptions, an RTPB tool did not lead to a statistically significant increase in fill rates overall, but it increased fill rates for the quartile of drug classes with the highest average out-of-pocket costs by 14.5 percentage points, with the largest gains among patients from the lowest-income communities served by the health system.

**Meaning:**

While the RTPB tool did not meaningfully increase fill rates overall, it was associated with higher fill rates for high-cost medications, particularly among patients from low-income communities, suggesting a potential role in reducing cost-related medication nonadherence in targeted contexts.

## Teaser

This post hoc analysis of a cluster randomized clinical trial aims to determine whether availability of a real-time prescription benefit tool increases prescription fill rates.

## Introduction

Rising drug prices and patient out-of-pocket cost burden contribute to growing medication nonadherence. For instance, 1 in 3 patients in the US reported not filling a prescription drug due to costs, and studies estimate that between 25% and 50% of prescriptions are never filled.^1,2,3,4,5^ Cost-related medication nonadherence leads to worse health outcomes, including higher rates of mortality and higher overall health care spending, and patients with lower income are at disproportionate risk for cost-related nonadherence.^6,7,8,9,10^

Real-time prescription benefit (RTPB) tools are an electronic health record (EHR)–integrated system with the potential to reduce cost-related nonadherence. When clinically suitable lower-cost alternatives are available, RTPB tools alert the clinician ordering the medication to the patient’s out-of-pocket cost for the medication and recommend clinically equivalent medication alternatives with lower out-of-pocket cost if available. RTPB tools have been shown to lower out-of-pocket costs, particularly in the highest-cost drug classes.^11^

Payers, policymakers, and health systems are increasingly investing in RTPB tools. Two-thirds of hospitals have adopted RTPB tools, and the Centers for Medicare & Medicaid Services now requires Medicare Part D plans to integrate with at least 1 RTPB tool.^12,13,14^ Despite this growing use, whether RTPB systems improve cost-related nonadherence is unclear. Previous studies reported that practices with RTPB tools had higher fill rates than those without, though they were observational and did not examine heterogeneity in effects by drug class or patient group.^15,16,17^

We previously reported results from a cluster randomized clinical trial of an RTPB tool in a large ambulatory network with a diverse patient population, which found reductions in patient out-of-pocket costs.^11^ Building on those findings, we conducted this post hoc analysis of the same trial to evaluate the effect of RTPB recommendations on prescription fill rates, an outcome not prespecified in the original protocol. We stratified analyses by average drug class out-of-pocket cost and patient zip code median income level.

## Methods

### Trial Design

The trial used a clustered design with 1:1 randomization of outpatient practice profiles stratified by specialty at NYU Langone Health. Each practice within the health system’s EHR (Epic Systems) was assigned a unique practice profile, which was the smallest grouping where the RTPB system settings could be adjusted for either the intervention (RTPB recommendations) or control (no RTPB recommendations). Clinicians within a practice profile shared a specialty. In multispecialty practice locations, clinicians were divided by specialty into different practice profiles, and clinicians across different practice locations (eg, 2 physical sites for a group of cardiologists) were in some cases grouped into the same practice profile. We use the term *practice* to refer to practice profile.

The trial protocol is available in Supplement 1. Analyses of primary outcomes specified in the protocol were published previously.^11^ In this post hoc secondary analysis of the trial data, we use the same analytic framework to examine impacts on medication fills, another clinically significant outcome.

The original trial report analyzed medication orders from January 13 through July 31, 2021. For this post hoc analysis, we extended the randomized follow-up period through December 31, 2021, during which cluster randomization remained in effect, to improve statistical power. This cluster randomized clinical trial encompassed a quality improvement initiative at NYU Langone Health and, therefore, did not undergo institutional review board review. Informed consent was not applicable because this was a quality improvement initiative using routinely collected clinical data. The study followed Consolidated Standards of Reporting Trials (CONSORT) reporting guidelines specific to cluster randomized trials.^18^

### Intervention

The intervention was implementation of the Surescripts RTPB system, an EHR-enabled system that recommends lower-cost, clinically equivalent alternatives to prescribers at the point of order. If eligible alternatives are available, the RTPB system notifies prescribers of the patient’s estimated out-of-pocket cost for the prescription being ordered and suggests up to 3 alternatives with lower out-of-pocket costs. Of note, when a prescriber orders a medication with an available generic equivalent, the EHR defaults to the generic formulation unless the prescriber specifies otherwise. As a result, the RTPB-estimated out-of-pocket cost and recommended alternatives are generally based on the generic formulation rather than the brand-name medication. Out-of-pocket cost estimates are patient specific, accounting for each patient’s pharmacy benefit manager (PBM) and benefit design factors such as formulary, co-payment, and deductibles. Recommended alternatives could be a different medication considered clinically equivalent, a greater days’ supply of the same medication, and/or a mail-order pharmacy. Therapeutic alternatives were determined by each patient’s PBM, and suggested alternatives could vary by patient based on costs and determinations of clinical equivalence. To decrease notification burden, in this trial, recommendations were only offered if the total out-of-pocket cost of the initial prescription was at least $5 and if out-of-pocket cost savings from recommended alternatives were at least $0.10/d (or $3 for a 30-day fill). In both intervention and control practices, the RTPB engine evaluated all medication orders in the background using these criteria. In control practices, however, savings thresholds were set high enough that recommendations were never displayed to clinicians. This allowed us to apply the same eligibility criteria to medication orders in intervention and control practices, even though recommendations were displayed only in intervention practices. Prescribers in the ambulatory network were informed about the phased implementation of the real-time prescription benefits system but were not made aware of their assignment to the intervention or control group during the trial period.

### Data Description

The analytic dataset was constructed using medication order and fill data from NYU Langone Health’s EHR system. For each prescription order, we captured the medication; days supply; order date; pharmacy type (mail order or retail); pharmacy identifier; drug class; patient’s medical record number (MRN), age, sex, zip code, and insurance type (Medicare, commercial insurance, Medicaid, or other); and ordering clinician’s National Provider Identifier (NPI), specialty, and practice. We also observed out-of-pocket cost for each order.

Using pharmacy medication dispense reports supplied by Surescripts in the EHR, we obtained data on prescription fills. Surescripts aggregates dispense records from participating pharmacies and PBMs, and therefore, fill data are limited to dispensing events captured through those contracted entities. For each prescription fill in the dispense report, we observed patient MRN, medication, order date, pharmacy identifier, and prescriber NPI. Because the EHR medication orders and dispense reports lack a common single identifier, we matched medication orders to prescription fills using a combination of patient MRN, medication, prescriber NPI, and ±28-day order date window. We also tested robustness to alternative matching algorithms by additionally requiring a match on pharmacy identification and by shortening the allowable window between order and prescription fill dates to 14 days and 1 day, respectively. We could not observe the complete set of pharmacies and PBMs that did vs did not contribute dispense data.

### Study Sample and Outcome

The study sample comprised medication orders eligible for RTPB recommendations among patients whose medication order data appeared in the dispense reports. For an order to be eligible for an RTPB recommendation, out-of-pocket cost information needed to be available, clinically equivalent alternatives needed to be available, and per the intervention setting, the total out-of-pocket cost for the order being initiated was required to be $5 or more, and the cost difference between available alternatives and the initial order was required to be $0.10 per day or higher.

Prescription fill information was considered available for a patient if an order for that patient ever appeared in the medication dispense data. To assess potential selection into the analytic sample, we compared observable characteristics of patients whose orders were included in the analytic sample with patients whose orders were eligible for RTPB recommendations but excluded because the patient did not appear in the dispense data. However, because this approach conditions the sample on patients filling at least 1 prescription, we conducted sensitivity analyses that removed this condition and analyzed all orders. In another sensitivity analysis, we limited the sample to medication orders sent to pharmacies whose pharmacy identifier appeared at least once in the dispense data, regardless of whether the patient had a dispensing record.

We estimated impacts of the intervention across the entire sample in aggregate as well as stratified by average out-of-pocket costs in a drug class. We divided drug classes into quartiles based on their drug class average out-of-pocket costs using data from medication orders in the control group only. The outcome was whether a medication order was filled.

### Statistical Analysis

We specified logistic regression models to assess the impact of RTPB recommendations on the outcome. The explanatory variable of interest indicated whether the order was placed in a practice assigned to the intervention, and model covariates included patient age category (18-40, 41-64, or ≥65 years), sex, and primary insurance type (commercial, Medicare, Medicaid, or other), as well as specialty and drug class fixed effects to account for differences in costs and drugs ordered by specialty and drug classes. Adjusting by specialty was important because practice sizes varied, creating some specialty imbalances despite stratified randomization. The main analyses clustered standard errors at the practice level, the unit of randomization.^19^ In sensitivity analyses, we also clustered at both the practice profile and patient levels using multiway clustering.

We performed several subgroup analyses. First, to assess whether impacts of the intervention differed by income, we stratified medication orders by the median household income in patients’ zip code as reported in the American Community Survey.^20^ We also stratified analyses by insurance type. Since patients receiving mail-order prescriptions may face fewer barriers to filling them, we also stratified medication orders based on whether they were mail-order prescriptions or filled in person. As a falsification test, we analyzed the impact of RTPB availability on fill rates for medication orders that were not subject to a recommendation, where no effect of the RTPB tool would be expected.

Statistical tests were 2-tailed, and *P* < .05 was considered statistically significant. We performed data analyses from October 18, 2022, to August 9, 2024, using R, version 4.2.1 (R Foundation for Statistical Computing).

## Results

**Figure 1.**
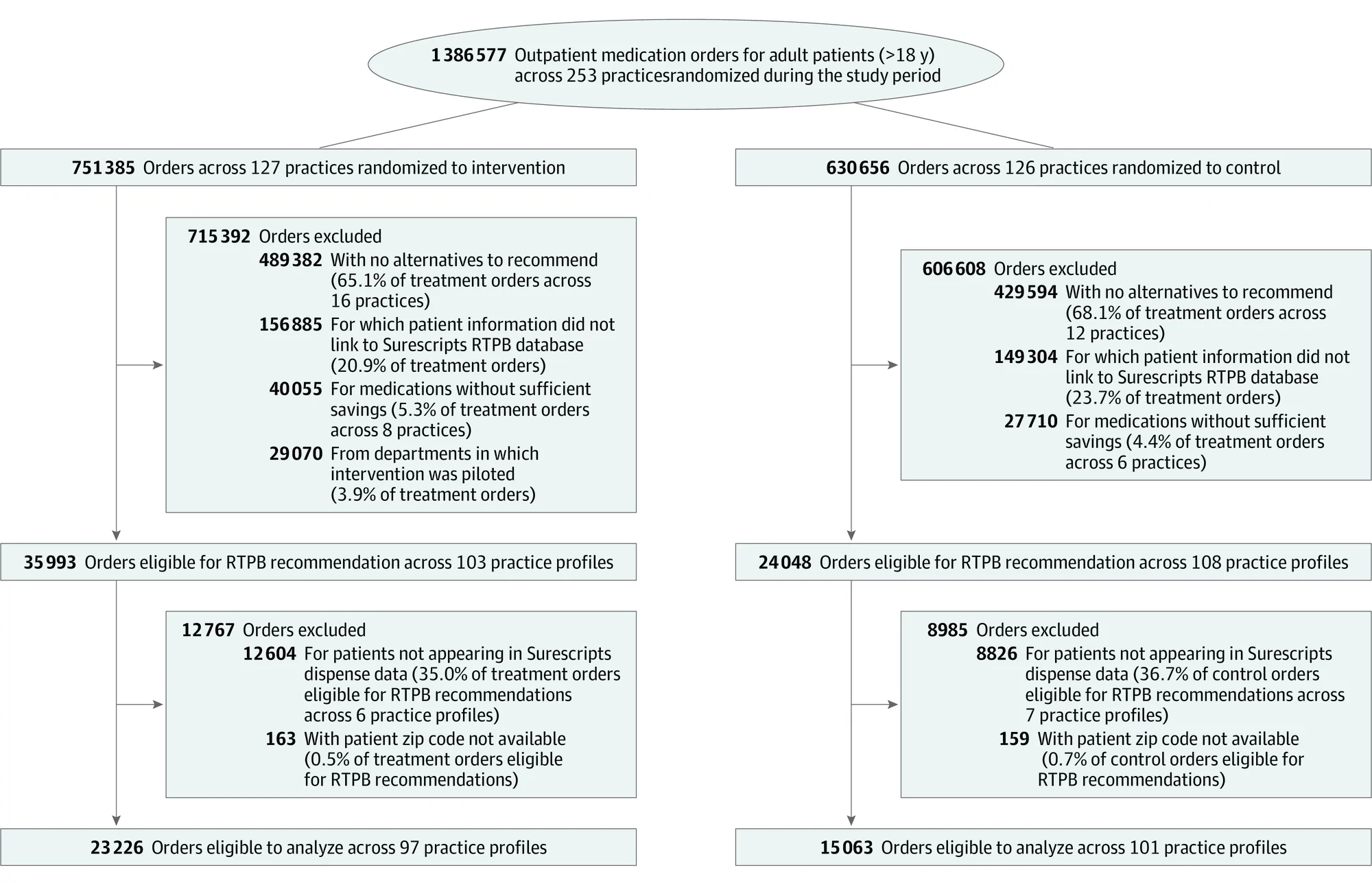
CONSORT Diagram Data were drawn from the electronic health record database. RTPB indicates real-time prescription benefit.

Of the 1 386 577 medication orders placed in the ambulatory setting during the study period, 38 289 (2.8%) were included in the analytic sample (Figure 1). Of all medication orders placed, 22.1% (intervention, 156 885 orders; control, 149 304 orders) were excluded because patient or PBM information did not link to the RTPB database, and 66.3% of orders (intervention, 489 382 orders; control, 429 594 orders) were excluded because they did not have lower-cost alternatives. Of the 60 041 orders eligible for RTPB recommendations, 38 289 orders (63.8%) were for patients whose data also appeared in dispense reports. Orders in the analytic dataset were similar across most dimensions to the broader set of orders placed during the study period at NYU Langone Health (eTable 1 in Supplement 2). However, one difference was that orders for Medicaid-enrolled patients were underrepresented in the analytic dataset because patients enrolled in Medicaid face minimal cost sharing for drugs. Observable patient characteristics were similar between medication orders included in the analytic sample and RTPB-eligible orders that were excluded because the patient did not appear in the dispense data (eTable 2 in Supplement 2).

Among the 38 289 medication orders in the analytic sample, characteristics between orders in the RTPB and control groups were similar (Table 1). Overall, 22 690 orders (59.3%) were for female patients, while 15 599 (40.7%) were for male patients, and 21 897 orders (57.2%) were for Medicare-insured patients, while 14 792 (38.6%) were for commercially insured patients. The median (IQR) annual income by zip code of residence was $101 340 ($75 341-$126 629). Imbalances in practice specialty were driven by differences in practice sizes across the intervention and control groups, underscoring the importance of adjusting for specialty in these analyses.

**Table 1.** Patient- and Practice-Level Characteristics of Medication Orders^a^

Characteristic	No. (%)		
	All	RTPB	Control
Total No.			
Orders	38 289	23 226	15 063
Patients	22 357	13 838	9458
Practices	198	97	101
Patient-level characteristics			
Sex			
Female	22 690 (59.3)	13 443 (57.9)	9247 (61.4)
Male	15 599 (40.7)	9783 (42.1)	5816 (38.6)
Age, y			
18-40	4759 (12.4)	3198 (13.8)	1561 (10.4)
41-64	10 876 (28.4)	6500 (28.0)	4376 (29.1)
≥65	22 654 (59.2)	13 528 (58.2)	9126 (60.6)
Insurance type			
Commercial	14 792 (38.6)	9162 (39.4)	5630 (37.4)
Medicaid	114 (0.3)	60 (0.3)	54 (0.4)
Medicare	21 897 (57.2)	13 039 (56.1)	8858 (58.8)
Other	1486 (3.9)	965 (4.2)	521 (3.5)
Zip-code level median income, $			
21 201-75 340	9573 (25.0)	6126 (26.4)	3447 (22.9)
75 341-101 339	9572 (25.0)	5826 (25.1)	3746 (24.9)
101 340-126 628	9572 (25.0)	5595 (24.1)	3977 (26.4)
126 629-250 001	9572 (25.0)	5679 (24.5)	3893 (25.8)
Practice specialty			
Primary care	14 000 (36.6)	9131 (39.3)	4869 (32.3)
Cardiology	6819 (17.8)	5554 (23.9)	1265 (8.4)
Neurology	2755 (7.2)	878 (3.8)	1877 (12.5)
Endocrinology	2293 (6.0)	1555 (6.7)	738 (4.9)
Gastroenterology	1483 (3.9)	311 (1.3)	1172 (7.8)
Surgery	1642 (4.3)	1157 (5.0)	485 (3.2)
Rheumatology	1623 (4.2)	137 (0.6)	1486 (9.9)
Mental health	1888 (4.9)	1765 (7.6)	123 (0.8)
Other	5786 (15.1)	2738 (11.8)	3048 (20.2)

Abbreviation: RTPB, real-time prescription benefit.

^a^ Data for orders were drawn from the NYU Langone Health system between January 13, 2021, and December 31, 2021. RTPB and control values may not sum to total due to a subset of patients and prescribers being exposed to the intervention and control.

Respectively, 55% and 54% of medication orders in the intervention and control groups were filled, with no observed impact of RTPB recommendations on fill rates (unadjusted difference: 0.2 percentage points [pp], 95% CI, −2.7 to 3.1 pp; adjusted difference: 1.2 pp; 95% CI, −1.3 to 3.7 pp) (Table 2). Among orders placed for drugs in the highest-cost drug classes (average out-of-pocket cost for 30-day supply of >$120.83), the fill rates for the intervention and control groups were 49% vs 33%, respectively, implying an unadjusted intervention effect of 16.1 pp (95% CI, 8.3-23.9 pp). After covariate adjustment, RTPB recommendations increased fill rates by 14.5 pp (95% CI, 8.4-20.6 pp) in the highest-cost drug classes. Among orders for lower-cost drug classes, fill rates were not impacted by RTPB recommendations. In the lowest-cost drug classes (average out-of-pocket cost for 30-day supply of <$22.60), the fill rates for the intervention and control groups were 59% vs 61%, respectively (unadjusted difference: −2.3 pp; 95% CI, −6.6 to 2.0 pp; adjusted difference: −0.6 pp; 95% CI, −3.9 to 2.7 pp). The most commonly ordered drug classes by cost quartile in the sample are reported in eTable 3 in Supplement 2.

**Table 2.** Associations Between Real-Time Prescription Benefit (RTPB) Availability and Medication Order Fill Rates, Overall and Stratified by Average Drug Class Out-of-Pocket Cost^a^

Variable	Total No.	Orders filled, %		Difference (95% CI), pp^b^	
		RTPB	Control	Unadjusted^c^	Adjusted^d^
All orders	38 289	55	54	0.2 (−2.7 to 3.1)	1.2 (−1.3 to 3.7)
Average out-of-pocket-cost in drug class for 30-d supply^e^					
>$120.83	1814	49	33	16.1 (8.3 to 23.9)	14.5 (8.4 to 20.6)
$50.11-$120.83	6798	47	46	0.9 (−5.2 to 7.0)	2.8 (−1.1 to 6.7)
$22.60-$50.10	10 924	52	54	−1.6 (−5.3 to 2.1)	0.5 (−2.8 to 3.8)
<$22.60	18 753	59	61	−2.3 (−6.6 to 2.0)	−0.6 (−3.9 to 2.7)

Abbreviation: pp, percentage point.

^a^ The order-level outcome was the proportion of orders filled. The analytic sample included medication orders comprising 2032 unique medications.

^b^ 95% CIs are based on heteroskedasticity-robust standard errors clustered at the level of randomization (the practice profile).

^c^ Estimated via logistic regression of the fill rate on an indicator for the intervention group with no other covariates.

^d^ Estimated via logistic regression of the fill rate, including the following covariates: indicators for specialty type, pharmaceutical class, age group (18-40, 41-64, and ≥65 years), sex, and insurance type (commercial, Medicaid, Medicare, or other).

^e^ Cost quartile was estimated by calculating the average out-of-pocket cost in each drug class for prescriptions in the control group and grouping drug classes into quartiles by average out-of-pocket cost for a 30-day supply.

**Figure 2.**
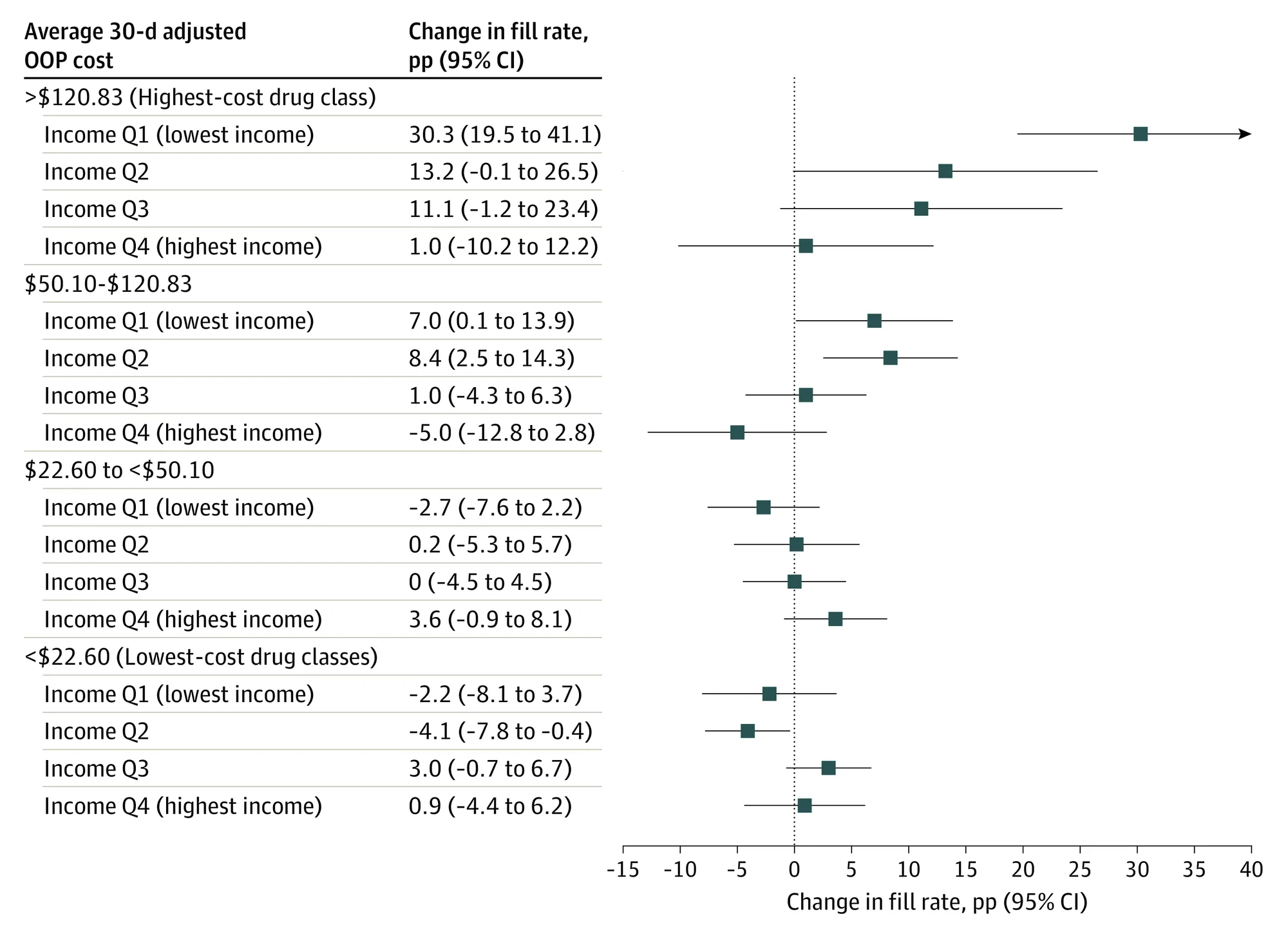
Dot Plot of Real-Time Prescription Benefit Availability and Prescription Fill Rates Stratified by Patient Area-Level Income and Average Out-of-Pocket (OOP) Cost in Medication Drug Class Data were drawn from the electronic health record database. Orders were divided into quartiles (Q) by calculating the average 30-day adjusted OOP cost for each drug pharmaceutical class using data from the control group. The sample was further stratified into income quartiles based on the median household income in the patient’s zip code using data from the American Community Survey. The raw proportion of orders filled by control and intervention group were calculated. Covariates include indicators for specialty and drug pharmaceutical class and patients’ age category, sex, and insurance type (private, Medicare, Medicaid, or other). Heteroskedasticity-robust standard errors clustered at the level of randomization were used to calculate the 95% CIs. pp Indicates percentage point.

When stratified by median household income by zip code, patients living in zip codes in the lowest quartile of median annual household income (≤$75 340) had the greatest increase in fill rates (Figure 2). Among the orders placed for drugs in the highest-cost drug classes, fill rates for patients in the lowest quartile of household income were 27% in the control group and 49% in the intervention group. RTPB recommendations led to an adjusted increase in fill rates of 30.3 pp (95% CI, 19.5-41.1 pp) in this income stratum. In other strata among patient groups with higher incomes, adjusted impacts of RTPB recommendations in high-cost drug classes were positive but not statistically significant (highest-income quartile: 1.0 pp; 95% CI, −10.2 to 12.2 pp). In contrast, among orders placed in the lowest-cost drug classes (average out-of-pocket cost for 30 days supply of <$22.60), fill rates for neither patients in the lowest income quartile nor other quartiles were impacted by RTPB recommendations. Estimates from sensitivity tests were similar to the main results. Results were also similar when using alternate algorithms to match medication order and fill data and when altering the date window on which medication orders and fills are matched (eTable 4 in Supplement 2). Results were also similar when removing filtering based on the patient appearing in the medication dispense data and when limiting the sample to orders at pharmacies that appeared in the medication dispense data (eTable 5 in Supplement 2). Effects of RTPB recommendations did not differ substantially between commercially and Medicare-insured patients (eTable 6 in Supplement 2). These results were similar when stratifying orders by mail-order status as well (eTable 7 in Supplement 2). Results were virtually unchanged when models were reestimated with 2-way clustering at the practice and patient levels (eTable 8 in Supplement 2). In the falsification test examining orders not subject to an RTPB recommendation, there were no statistically significant differences in fill rates between intervention and control groups (eTable 9 in Supplement 2).

## Discussion

An RTPB tool implemented in a large ambulatory network did not meaningfully increase prescription fill rates overall among medication orders eligible for recommendations. However, RTPB recommendations increased fill rates within high-cost drug classes, with the largest effects among patients in the lowest income zip codes, who had the lowest baseline fill rates.

These findings indicate that for medications in high-cost drug classes (for which baseline fill rates were lowest) and for patients with lower income, RTPB tools may mitigate cost-related drug nonadherence. The highest-cost drug classes included antiretrovirals, antimigraine medications, coronary vasodilators, and selected antibiotics. Previous research has found that cost-related medication nonadherence is associated with worse health outcomes, suggesting that increased fills for high-cost medications may have important clinical implications. Although RTPB tools reduced out-of-pocket costs for less costly medications, the absence of corresponding improvements in fill rates indicates that effects on prescription fills were either too small to detect or that nonadherence in these drug classes was due to factors unrelated to costs.

The RTPB tool had the largest benefit for patients from the lowest-income communities and, therefore, may help address existing disparities in medication adherence. Previous studies have found greater price sensitivity among patients with lower income and that these populations particularly benefit from increased engagement with their prescribers.^21,22^ RTPB recommendations may facilitate cost conversations between prescribers and patients. Because this study was conducted in the New York City metropolitan area, the lowest-income group in the sample may have had higher incomes than populations with low income in other parts of the US. Therefore, RTPB recommendations could have equal or larger effects in settings where patients have fewer financial resources.

These findings suggest that efforts to expand and refine use of RTPB tools may be most valuable for patients facing higher out-of-pocket costs rather than as a universal strategy to increase prescription fills. RTPB recommendations were associated with meaningful differences in fill rates among patients with lower income who were prescribed the highest-cost medications, even though this group represented less than 5% of the medication orders evaluated. This targeted reach partially reflects the specific configuration of the RTPB tool in this health system, where recommendations were displayed only when the initiated order and available alternatives met prespecified cost and savings thresholds. The reach and impact of RTPB tools may depend on how they are configured and targeted to prescriptions where cost information is most likely to affect filling behavior. Notably in this study, a small fraction of medication orders were eligible for RTPB recommendations.^16,17^ Although many drugs may lack alternatives, there was substantial variation across PBMs in whether an alternative was recommended for a medication. As RTPB requirements expand, these findings suggest that policy and implementation efforts should focus not only on adoption, but also on whether RTPB tools identify clinically appropriate, lower-cost alternatives for the prescriptions where cost barriers are most likely to affect filling behavior.

RTPB tools are unique in their effectiveness given that other price transparency tools have had limited success.^23,24^ RTPB tools may be more effective than consumer-facing transparency tools because they target clinicians and provide actionable recommendations at the point of decision-making. These features may better facilitate cost-of-care conversations and shared decision-making.^25,26^

### Limitations

This work had several limitations. First, the trial was limited to 1 academic health system and 1 RTPB tool. Results may not generalize to other populations or RTPB systems. Nonetheless, we note that NYU Langone Health encompasses a large ambulatory network with a diverse population, and Surescripts is the largest e-prescribing network in the country. Second, the RTPB tool was limited in the proportion of medication orders that were eligible for RTPB recommendations. Other RTPB implementation practices may experience different outcomes, with potentially greater benefit if RTPB tools were more broadly applied. Third, we conducted multiple stratified analyses, which increases the potential for type I error; these results should be interpreted as exploratory and hypothesis generating. Finally, prescription fills were measured using dispense reports in the EHR, which capture prescription fills from participating pharmacies and PBMs but not all dispensing events. We could not compare contributing vs noncontributing entities, but included patients were similar across observable EHR characteristics to otherwise eligible excluded patients. In addition, because RTPB availability was randomized across practices, incomplete capture of dispensing events is unlikely to differ systematically between intervention and control practices.

## Conclusions

In this post hoc analysis of a cluster randomized clinical trial, RTPB recommendations improved medication fill rates for high-cost drug classes, though overall fill rates were not increased. The RTPB tool held the greatest benefit for patients with the lowest income, highlighting the potential of RTPB recommendations and price transparency more generally in improving equity in the health care system.

## Notes

Supplement 1. Trial Protocol

Supplement 2. **eTable 1. **Comparison of all outpatient orders in NYU Langone system v. orders in analytic sample during study period **eTable 2.** Characteristics of RTPB-eligible medication orders excluded from the analytic sample **eTable 3.** Five most frequently ordered drug classes by average out-of-pocket cost quartile **eTable 4.** Associations between RTPB availability and prescription fill rates: sensitivity analyses using alternate merging criteria for medication dispense data **eTable 5.** Effects of RTPB recommendations on prescription fill rates: sensitivity analyses (1) removing conditioning on patient appearing in medication dispense data and (2) conditioning on pharmacy appearing in dispense data **eTable 6.** Associations between RTPB availability and prescription fill rates: sensitivity analyses stratified by insurance type **eTable 7.** Associations between RTPB availability and prescription fill rates: sensitivity analyses stratified by (a) non-mail order and (b) mail order prescriptions **eTable 8.** Associations between RTPB availability and prescription fill rates: sensitivity analysis using two-way clustering at the practice and patient levels **eTable 9.** Falsification analysis: orders not eligible for RTPB recommendations

Supplement 3. Data Sharing Statement

## References

[1] Doshi JA, Li P, Huo H, Pettit AR, Armstrong KA. Association of patient out-of-pocket costs with prescription abandonment and delay in fills of novel oral anticancer agents. J Clin Oncol. 2018;36(5):476-482. doi:10.1200/JCO.2017.74.5091 29261440

[2] Gleason PP, Starner CI, Gunderson BW, Schafer JA, Sarran HS. Association of prescription abandonment with cost share for high-cost specialty pharmacy medications. J Manag Care Pharm. 2009;15(8):648-658. doi:10.18553/jmcp.2009.15.8.648 19803554 PMC10441203

[3] Fischer MA, Stedman MR, Lii J, . Primary medication non-adherence: analysis of 195,930 electronic prescriptions. J Gen Intern Med. 2010;25(4):284-290. doi:10.1007/s11606-010-1253-9 20131023 PMC2842539

[4] Fischer MA, Choudhry NK, Brill G, . Trouble getting started: predictors of primary medication nonadherence. Am J Med. 2011;124(11):1081.e9-1081.e22. doi:10.1016/j.amjmed.2011.05.028 22017787

[5] Fischer MA, Choudhry NK, Bykov K, . Pharmacy-based interventions to reduce primary medication nonadherence to cardiovascular medications. Med Care. 2014;52(12):1050-1054. doi:10.1097/MLR.0000000000000247 25322157

[6] Wagner TH, Heisler M, Piette JD. Prescription drug co-payments and cost-related medication underuse. Health Econ Policy Law. 2008;3(pt 1):51-67. doi:10.1017/S1744133107004380 18634632

[7] Dusetzina SB, Besaw RJ, Whitmore CC, . Cost-related medication nonadherence and desire for medication cost information among adults aged 65 years and older in the US in 2022. JAMA Netw Open. 2023;6(5):e2314211. doi:10.1001/jamanetworkopen.2023.14211 37200029 PMC10196872

[8] Chandra A, Flack E, Obermeyer Z. The health costs of cost-sharing. National Bureau of Economic Research working paper 28439. February 2021. Accessed July 13, 2026. https://www.nber.org/system/files/working_papers/w28439/revisions/w28439.rev0.pdf

[9] Pittman DG, Tao Z, Chen W, Stettin GD. Antihypertensive medication adherence and subsequent healthcare utilization and costs. Am J Manag Care. 2010;16(8):568-576. 20712390

[10] Chang HY, Kan HJ, Shermock KM, Alexander GC, Weiner JP, Kharrazi H. Integrating e-prescribing and pharmacy claims data for predictive modeling: comparing costs and utilization of health plan members who fill their initial medications with those who do not. J Manag Care Spec Pharm. 2020;26(10):1282-1290. doi:10.18553/jmcp.2020.26.10.1282 32996394 PMC10391092

[11] Desai SM, Chen AZ, Wang J, . Effects of real-time prescription benefit recommendations on patient out-of-pocket costs: a cluster randomized clinical trial. JAMA Intern Med. 2022;182(11):1129-1137. doi:10.1001/jamainternmed.2022.3946 36094537 PMC9468947

[12] Modernizing Part D and Medicare Advantage to Lower Drug Prices and Reduce Out-of-Pocket Expenses. 42 CFR §422, 423 (2019). Accessed February 7, 2024. https://www.federalregister.gov/documents/2019/05/23/2019-10521/modernizing-part-d-and-medicare-advantage-to-lower-drug-prices-and-reduce-out-of-pocket-expenses

[13] Hospital adoption of real-time benefit tools. Office of the National Coordinator for Health Information Technology. Updated April 2026. Accessed July 13, 2026. https://www.healthit.gov/data/quickstats/hospital-adoption-real-time-benefit-tools/

[14] Klebanoff MJ, Li P, Chatterjee P, Doshi JA. Real-time prescription benefit tool adoption among US hospitals. JAMA Health Forum. 2024;5(10):e243181. doi:10.1001/jamahealthforum.2024.3181 39422890 PMC11581665

[15] Bhardwaj S, Merrey JW, Bishop MA, Yeh HC, Epstein JA. Impact of real-time benefit tools on patients’ access to medications: a retrospective cohort study. Am J Med. 2022;135(11):1315-1319.e2. doi:10.1016/j.amjmed.2022.06.017 35896143

[16] Reise R, Ndai AM, Dewar MA, Schentrup AM, Yang J, Vouri SM. Assessment of the utilization of real-time prescription benefits for patient cost savings within an outpatient setting. Explor Res Clin Soc Pharm. 2024;14:100460. doi:10.1016/j.rcsop.2024.100460 38974055 PMC11227025

[17] Fitts A, Teare AJ, Nelson SD. Price transparency at the point of prescribing with real-time prescription benefits. Am J Health Syst Pharm. 2024;81(19):e620-e626. doi:10.1093/ajhp/zxae108 38713809 PMC11419340

[18] Hopewell S, Chan AW, Collins GS, . CONSORT 2025 statement: updated guideline for reporting randomized trials. JAMA. 2025;333(22):1998-2005. doi:10.1001/jama.2025.4347 40228499

[19] Abadie A, Athey S, Imbens GW, Wooldridge JM. When should you adjust standard errors for clustering? Q J Econ. 2023;138(1):1-35. doi:10.1093/qje/qjac03838414485

[20] American Community Survey data. US Census Bureau. Accessed February 9, 2024. https://www.census.gov/programs-surveys/acs/data.html

[21] Chen A, Lakdawalla DN. Healing the poor: the influence of patient socioeconomic status on physician supply responses. J Health Econ. 2019;64:43-54. doi:10.1016/j.jhealeco.2019.02.001 30797112 PMC6481618

[22] Mishra SI, Gioia D, Childress S, Barnet B, Webster RL. Adherence to medication regimens among low-income patients with multiple comorbid chronic conditions. Health Soc Work. 2011;36(4):249-258. doi:10.1093/hsw/36.4.249 22308877 PMC3606079

[23] Mummadi SR, Mishra R. Effectiveness of provider price display in computerized physician order entry (CPOE) on healthcare quality: a systematic review. J Am Med Inform Assoc. 2018;25(9):1228-1239. doi:10.1093/jamia/ocy076 29982523 PMC7646898

[24] Langley T, Lacey J, Johnson A, . An evaluation of a price transparency intervention for two commonly prescribed medications on total institutional expenditure: a prospective study. Future Healthc J. 2018;5(3):198-202. doi:10.7861/futurehosp.5-3-198 31098566 PMC6502609

[25] Lalani HS, Tessema FA, Kesselheim AS, Rome BN. Availability and cost of expensive and common generic prescription drugs: a cross-sectional analysis of direct-to-consumer pharmacies. J Gen Intern Med. 2024;39(12):2187-2195. doi:10.1007/s11606-024-08623-y 38321315 PMC11347552

[26] Lalani HS, Hwang CS, Kesselheim AS, Rome BN. Strategies to help patients navigate high prescription drug costs. JAMA. 2024;332(20):1741-1749. doi:10.1001/jama.2024.17275 39432312

