# Error in Funding/Support

**DOI:** 10.1001/jamahealthforum.2026.3963

**Published:** 2026-09-25

In the Original Investigation titled “Real-Time Prescription Benefit Tool Availability and Prescription Medication Fill Rates: A Post Hoc Analysis of a Cluster Randomized Clinical Trial,”^1^ published August 14, 2026, the name of one of the funders was listed incorrectly in the Funding/Support section. The correct name of the funder is the NCPDP Foundation. This article has been corrected.

## References

[1] Ying R, Padmanabhan P, Szerencsy A, Mehrotra A, Horwitz LI, Desai SM. Real-time prescription benefit tool availability and prescription medication fill rates: a post hoc analysis of a cluster randomized clinical trial. JAMA Health Forum. 2026;7(8):e262739. doi:10.1001/jamahealthforum.2026.2739 42599729 PMC13476843

