# Supplementary material for: Real-Time Prescription Benefit Tool Availability and Prescription Medication Fill Rates: A Post Hoc Analysis of a Cluster Randomized Clinical Trial
Source: JAMA Health Forum. 2026 Aug 14;7(8):e262739. doi: 10.1001/jamahealthforum.2026.2739 (PMC13476843; doi:10.1001/jamahealthforum.2026.2739)
Supplement: Supplementary file 1 [file jamahealthforum-e262739-s001.pdf]

Real-Time Benefits Physician-Targeted Price Transparency: A Cluster Randomized Intervention

Pre-specified Analysis Plan

First version: November 2021

Revised: January 2021

1. Introduction

The goal of this study is to evaluate whether presenting patient out-of-pocket costs to a provider at the time of prescribing leads to prescribing of medications with lower out-of-pocket (OOP) costs. This document details our analysis plan for a randomized controlled trial that will study the impact of a real time prescription benefits check system and was prepared before we viewed any unblinded study data.

We also anticipate that other outcomes or analyses inspired by the main results may be pursued in the future, and such future analyses we hope to pursue are also documented here.

The structure of the plan is as follows. Section 2 describes the intervention, Section 3 describes the randomization, and Section 4 describes planned analyses.

2. The intervention

The goal of the real-time benefits check (RTBC) tool is to facilitate prescribing of lower-cost alternatives by providing patient out-of-pocket cost information to physicians at the point of prescribing. RTBC is implemented through a partnership between Surescripts and Epic.

For a subset of prescriptions ordered, RTBC provides physicians with information about patient out-of-pocket (OOP) cost for medications at the point of outpatient prescribing. OOP is inclusive of any copay, coinsurance, and deductible that the patient owes given their prescription drug benefit plan (see screenshot in Figure below). If the physician is submitting a prescription order and a clinically-appropriate alternative with a lower out-of-pocket cost is available, an alert with out-of-pocket cost information for the drug being ordered as well as up to three lower-cost alternatives will be displayed. The physician can then prescribe the original drug or one of the alternative drugs. Unless the physician specifies that the prescription should be dispensed as written, all orders default to a generic. As such, the out-of-pocket costs for the generic version will be displayed.

Information on patient OOP costs for the drug being prescribed and any available alternatives is only available when the patient's identifying information and prescription benefits in Epic can be matched to the Surescripts database. Therefore, our analytic data will comprise orders for which OOP costs were available.

Alerts will be displayed if alternatives with sufficient out-of-pocket savings are available. Specifically, alerts will be displayed if the requested medication has an out-of-pocket price per fill of >$5.00 and a lower-cost alternative with savings of at least $0.10/day is available. Suggested alternatives may include lower-cost drugs deemed to be clinically-appropriate substitutes, the same drug from a mail-order pharmacy, or the same drug in a different quantity. The control group of physicians will not be shown such an alert even when a lower-cost alternative is available.

The intervention will begin on November 18, 2020 4PM.

Update: In January 2021 it was determined that due to an IT error, the intervention had only been turned on for half of providers in the original intervention group. This was rectified and the intervention was turned on for all physicians in the intervention group on January 13,2021. As a result of this, we revised our analysis plan to start on January 13, 2021 when the RTBC system was enabled for all intervention providers.

Prior to implementation, a written orientation and guide to the RTBC will be sent to all NYU Langone-employed physicians as part of a weekly email series on Epic updates. The contents of this email which includes a snapshot of an alert are shown below.

Real Time Benefit Checking for Epic Medication Orders

Expansion of Real Time Benefit Checking (RTBC) Pilot in Epic

The Real Time Benefit Checking pilot in Epic will expand throughout NYULH this month. This functionality allows many departments to view out of pocket costs for medications at the time of ordering and easily select therapeutically equivalent, lower cost alternatives. In collaboration with MCIT and the Department of Population Health, the Clinically Integrated Network (CIN) will be analyzing RTBC adherence outcomes and savings for providers, patients and health plans.

Key points of Real Time Benefit Checking:

- **Saves Time** - Currently patients find out that drug is not covered or is too costly after they arrive at the pharmacy. This results in call back messages to the providers and additional work to prescribe an alternative medication. Epic RTBC can help prevent this situation with no additional work for providers or front office staff.
- **Saves Money** - Patients have the opportunity to be prescribed the least costly formulary alternative, which results in lower costs for both patients and health plans. Health plan savings translates to shared savings with CIN providers.
- **Better Adherence** – Patients are more likely to refill their medications when they can afford them.
- **Improves Clinical Outcomes** – Improved medication adherence results in goal attainment.
- **Improves Satisfaction** – Patients, providers and office staff will be happy with improvements in workflow efficiency and cost savings.

The CDC reports that 47% of individuals under age 65 with private insurance are enrolled in a high-deductible plans.[1] A survey by Kaiser Family Foundation revealed that high prescription drug costs leads to medication non-adherence and adverse health outcomes.[2] **It is estimated that 40% of prescribers will prescribe a lower cost alternative at time of prescribing which can save up to $130 per prescription.**[3]

In the proton pump inhibitor example below, your patient could pay $60 for a month supply of Dexilant or low as $13 for a three-month supply of omeprazole or pantoprazole from their mail order pharmacy.

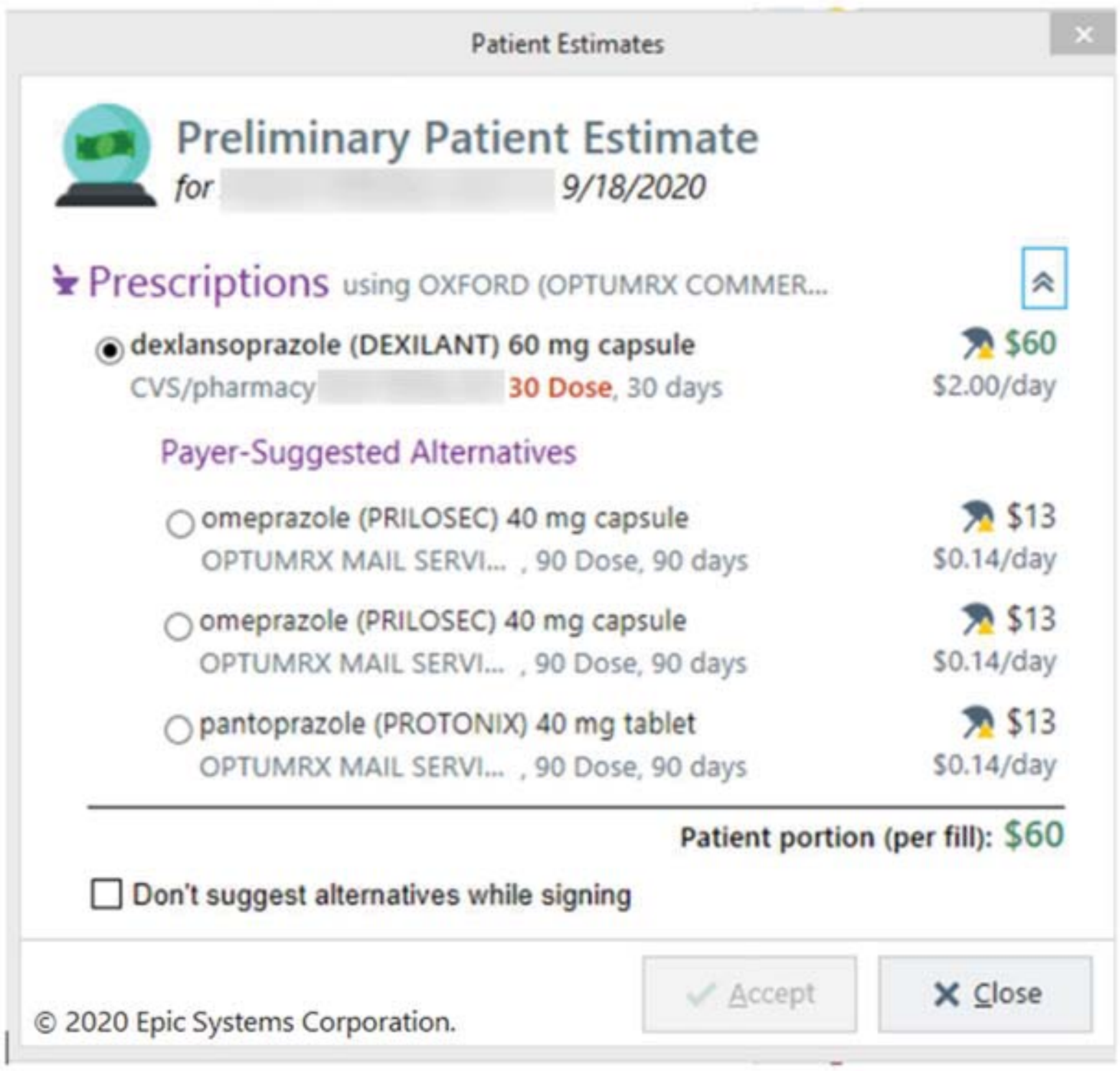

1.Cohen RA, Martinez ME, Zammitti EP, et al. Health Insurance Coverage: Early Release of Estimates from the National Health Interview Survey, Jan-Mar 2018. CDC. 8.18. https://www.cdc.gov/nchs/data/nhis/earlyrelease/insur201808.pdf

2.Kaiser Family Foundation Health Tracking Poll: – February 2019: Prescription Drugs. March 1, 2019.https://www.kff.org/health-reform/poll-finding/kff-health-tracking-poll-february-2019-prescription-drugs/

3.Proven Savings with Real-Time Benefits: The Key Components of a Solution that Delivers Results. Insightsfeature. 8/22/18. CVSHealth

4.Real-Time Benefit: Validating outcomes and Savings for Health Plans, Providers, and Patients. White Paper. SS&C Health. 2019. health.ssctech.com.

3. Randomization

We will randomize the implementation of the RTBC system across NYU's Faculty Group Practice and Family Health Centers.

We will randomize physician profiles to RTBC. Within a given practice location, there may be several departments which are specialty-specific. In the context of NYU, we translated departments into profiles. Profiles are groupings of departments designated by NYU Langone Health's Information Technology department for the purposes of IT-related implementations. Within a single physical practice site, there may be several profiles.

We used a stratified randomization technique, stratifying at the specialty level since the volume, average price, and opportunity for savings through prescribing of lower-cost drugs may vary across specialties. We grouped similar specialties together to ensure sufficient sample size within a given specialty. Balance between the treatment and control groups will be assessed on the number of drug orders, the number of prescribers, and the number of departments.

Some physicians may practice across multiple practices or departments. We do not anticipate concerns about contamination, because the intervention is at the order-level, and the same drug can have different out-of-pocket costs depending on a patient's benefit design and deductible.

4. Planned analysis

Data and outcomes. Data for our analysis will be drawn from NYU Langone's Epic electronic health record system. Epic data contains information about the prescription drug order, patient, and the prescribing physician. For every order in our sample, we can observe the medication that was prescribed, the date and time of prescribing, the quantity and days supply, whether the prescription required prior authorization, whether the prescription was specified to be dispensed as written, the pharmacy of the ordered drug (including mail-order pharmacy), and similar information for any alternatives available. Information about the prescriber and their department are also available. Our data also contains patient-level information including demographic characteristics, zip code, and patient's primary payer for medical and prescription drug benefits.

Out-of-pocket cost information is available when the patient information in Epic can be matched to the Surescripts database, the prescription drug manager (PBM) is identified and verified in the patient's file, and Surescripts has a contract with the patient's PBM.

Our analytic data set will comprise prescriptions for which out-of-pocket information was captured and alternatives were available with sufficient opportunities for savings to cause an alert to be displayed ($0.10/day in OOP).

Our primary outcome is patient out-of-pocket cost per order.

We will examine several secondary analyses. We will also test for changes in the likelihood that an order is placed to a mail-order pharmacy and the days supply. Another secondary outcome we will examine is overall spending on a fill using external data on drug costs as a proxy for actual payment which is not fully available in our data. To test for changes in the quantity of prescribing, we will examine the number of orders per visit.

Among the intervention group, we will measure the rate of alerts and the acceptance rates for alerts.

We will conduct several subgroup analyses as well.

The following secondary outcomes: likelihood of an order to a mail-order pharmacy and days supply. These will include whether an order was sent to a mail-in pharmacy and whether an alternative was selected when available.

We will conduct several stratified analyses to test for evidence of lower-cost prescribing in specific settings. First, we will conduct stratified analyses on drug classes where there are large opportunities for savings from switching to lower-cost drugs. OOP data across drugs generated once the RTBC system is turned on will be used to determine drug classes with

high prices and variation. Second, we will conduct analyses on high-OOP drugs, which will be empirically identified from price data generated from the RTBC system. We will subset the data to orders for branded drugs. We will stratify by new vs. continuing medications. We will also conduct subanalyses among orders for low-income patients. Low-income patients will be defined as patients residing in zip codes for which average income of residents is in the bottom quartile.

We will separately examine practices that are Family Health Centers, which are federally qualified health centers and predominantly treat Medicaid patients who face low cost sharing obligations for drugs.

We will examine the rate at which providers in the intervention group selected an alternative drug when an alert was displayed.

Statistical analysis. To evaluate whether the availability of real time benefits checks changes prescribing behavior, we will estimate the following models

$$(1) \qquad Outcome_{ijds} = f\left(\beta_0 + \beta_1 treat_d + \varepsilon_{ijds}\right)$$
$$(2) \qquad Outcome_{ijds} = f\left(\beta_0 + \beta_1 treat_d + \gamma X_{ij} + \varepsilon_{ijds}\right)$$
$$(3) \qquad Outcome_{ijds} = f(\beta_0 + \beta_1 treat_d + \gamma X_{ij} + \alpha_s + \varepsilon_{ijds})$$

where $Outcome_{ijpd}$ is the outcome for order i for patient j prescribed in department d in specialty s, $treat_{ipd}$ denotes whether that prescription was ordered in a department randomized to the treatment, X_j is a vector of patient-level factors (age, gender, whether the patient is insured by Medicaid, Medicare, or a commercial insurer), $\propto_s$ is a vector of specialty fixed effects, and ε_{irps} is an individual error term. For the main outcome, we will specify a log-linear model since out-of-pocket cost is likely to have a skewed distribution. In the case that examination of the distributional characteristics of our data suggests an alternate functional form would be more appropriate, we will specify the model as such. Standard errors will be clustered at the department level.

Potential future analyses. If approved, we will also conduct an online survey across physicians to examine whether the availability of RTBC information led to increased consideration of cost information when prescribing or greater engagement in cost-of-care conversations.

In the future, we also hope to examine the impacts of the likelihood of a patient filling a medication order and adherence.

Power analysis and implied intervention length.

We based our power calculation on a blinded pilot sample of our data drawn after the start of the intervention. This data informed the sample mean, standard deviation, intraclass correlation, and the number of orders with alternatives in a day. The latter metric allows us

to estimate how long we need to run the intervention. Since the sample was blinded, we took the sample mean as the control group mean for the purposes of our power calculation.

With a mean outcome of $1.67, and intraclass correlation within departments of 0.02, powering for an effect size of a 10% reduction in out-of-pocket costs with 5% significance level and 80% power, our power calculation suggests required sample size of 28,221 orders with alternatives. According to our pilot data, 190 orders with alternatives are placed each business day within the NYU system. This suggests we need to run our intervention for 148 days to arrive at a sample that facilitates a sufficiently powered analysis.
