# Supplementary material for: Real-Time Prescription Benefit Tool Availability and Prescription Medication Fill Rates: A Post Hoc Analysis of a Cluster Randomized Clinical Trial
Source: JAMA Health Forum. 2026 Aug 14;7(8):e262739. doi: 10.1001/jamahealthforum.2026.2739 (PMC13476843; doi:10.1001/jamahealthforum.2026.2739)
Supplement: Supplementary file 2 [file jamahealthforum-e262739-s002.pdf]

Supplemental Online Content

Ying R, Padmanabhan P, Szerencsy A, Mehrotra A, Horwitz LI, Desai SM. Real-time prescription benefit tool availability and prescription medication fill rates: a post hoc analysis of a cluster randomized clinical trial. *JAMA Health Forum*. 2026;7(8):e262739. doi:10.1001/jamahealthforum.2026.2739

eTable 1. Comparison of all outpatient orders in NYU Langone system v. orders in analytic sample during study period
eTable 2. Characteristics of RTPB-eligible medication orders excluded from the analytic sample
eTable 3. Five most frequently ordered drug classes by average out-of-pocket cost quartile
eTable 4. Associations between RTPB availability and prescription fill rates: sensitivity analyses using alternate merging criteria for medication dispense data
eTable 5. Effects of RTPB recommendations on prescription fill rates: sensitivity analyses (1) removing conditioning on patient appearing in medication dispense data and (2) conditioning on pharmacy appearing in dispense data
eTable 6. Associations between RTPB availability and prescription fill rates: sensitivity analyses stratified by insurance type
eTable 7. Associations between RTPB availability and prescription fill rates: sensitivity analyses stratified by (a) non-mail order and (b) mail order prescriptions
eTable 8. Associations between RTPB availability and prescription fill rates: sensitivity analysis using two-way clustering at the practice and patient levels
eTable 9. Falsification analysis: orders not eligible for RTPB recommendations

This supplemental material has been provided by the authors to give readers additional information about their work.

© 2026 Ying R et al. *JAMA Health Forum*.

eTable 1. Comparison of all outpatient orders in NYU Langone system v. orders in analytic sample during study period[a]

	All orders during study period	Orders in analytic sample
No. orders	1,386,577	38,289
No. patients	391,034	22,357
No. practices	254	198
**Order-level characteristics, No. (%)**		
Female	833,139 (60.1)	22,690 (59.3)
Age		
18-40	251,075 (18.1)	4,759 (12.4)
41-65	585,182 (42.2)	10,876 (28.4)
66+	550,320 (39.7)	22,654 (59.2)
Insurance type		
Commercial	571,402 (41.2)	14,792 (38.6)
Medicaid	229,987 (16.6)	114 (0.3)
Medicare	525,722 (37.9)	21,897 (57.2)
Others	59,461 (4.3)	1,486 (3.9)
Zip-code level population income		
< $75,340	344,352 (25.0)	9,573 (25.0)
$75,340 - $101,339	344,352 (25.0)	9,572 (25.0)
$101,339 - $126,628	344,351 (25.0)	9,572 (25.0)
> $126,628	344,351 (25.0)	9,572 (25.0)
Specialty		
Primary Care	583,788 (42.2)	14,000 (36.6)
Cardiology	179,637 (13.0)	6,819 (17.8)
Neurology	68,085 (4.9)	2,755 (7.2)
Endocrinology	62,814 (4.5)	2,293 (6.0)
Gastroenterology	55,036 (4.0)	1,483 (3.9)
Surgery	77,849 (5.6)	1,642 (4.3)
Rheumatology	53,700 (3.9)	1,623 (4.2)
Mental Health	43,106 (3.1)	1,888 (4.9)
Others	262,562 (18.8)	5,786 (15.1)
Out-of-pocket cost (for orders with available data), $	$33.32	$53.35

[a]Data for orders were drawn from the NYU Langone Health system between January 13, 2021, and December 14, 2021. RTPB and control values may not sum to total due to a subset of patients and prescribers being exposed to the intervention and control.

© 2026 Ying R et al. *JAMA Health Forum*.

eTable 2. Characteristics of RTPB-eligible medication orders excluded from the analytic sample

	All	RTPB	Control
No. orders	21205	12481	8724
No. patients	15,150	8,995	6,460
No. practices	196	98	98
**Order-level characteristics, No. (%)**			
Female	12307 (58.0)	7102 (56.9)	5205 (59.7)
Age			
18-40	3139 (14.8)	1850 (14.8)	1289 (14.8)
41-65	6182 (29.2)	3570 (28.6)	2612 (29.9)
66+	11884 (56.0)	7061 (56.6)	4823 (55.3)
Insurance type			
Commercial	8833 (41.7)	5160 (41.3)	3673 (42.1)
Medicaid	43 (0.2)	22 (0.2)	21 (0.2)
Medicare	11448 (54.0)	6757 (54.1)	4691 (53.8)
Others	881 (4.2)	542 (4.3)	339 (3.9)
Zip-code level median income			
$21,201 - $75,340	6029 (28.4)	3589 (28.8)	2440 (28.0)
$75,341 - $101,339	5272 (24.9)	3131 (25.1)	2141 (24.5)
$101,340 - $126,628	4868 (23.0)	2767 (22.2)	2101 (24.1)
$126,629 - $250,001	5036 (23.7)	2994 (24.0)	2042 (23.4)
Specialty			
Primary Care	6691 (31.6)	4159 (33.3)	2532 (29.0)
Cardiology	3867 (18.2)	3230 (25.9)	637 (7.3)
Neurology	1495 (7.1)	536 (4.3)	959 (11.0)
Endocrinology	1424 (6.7)	988 (7.9)	436 (5.0)
Gastroenterology	1207 (5.7)	261 (2.1)	946 (10.8)
Surgery	1104 (5.2)	688 (5.5)	416 (4.8)
Rheumatology	884 (4.2)	74 (0.6)	810 (9.3)
Mental Health	577 (2.7)	548 (4.4)	29 (0.3)
Others	3956 (18.6)	1997 (16.0)	1959 (22.5)

© 2026 Ying R et al. *JAMA Health Forum*.

eTable 3. Five most frequently ordered drug classes by average out-of-pocket cost quartile

	Pharmacologic Class	Mean OOP	% of orders in quartile	No. orders
Average out-of-pocket-cost in medication drug class for 30 days supply:				
>$120.8 (Highest cost drug classes)	cephalosporin antibiotics - 3rd generation	137.53	14%	252
	antivirals, hiv-spec, nucleoside-nucleotide analog	230.61	9%	160
	antimigraine preparations	325.7	9%	158
	vasodilators,coronary	89.08	6%	106
	laxatives and cathartics	814.11	6%	104
$50.1-$120.8	adrenergics, aromatic, non-catecholamine	67.18	14%	948
	lipotropics	66.96	10%	669
	tetracycline antibiotics	78.93	8%	549
	antivirals, general	69.77	7%	483
	anti-obesity glucagon-like peptide-1 recep agonist	77.24	5%	360
$22.6-$50.1	thyroid hormones	23.93	23%	2465
	proton-pump inhibitors	27.8	13%	1409
	anticonvulsants	40.55	11%	1172
	skeletal muscle relaxants	24.04	5%	549
	beta-adrenergic agents, inhaled, short acting	33.94	5%	525
<$22.6 (Lowest cost drug classes)	antihyperlipidemic-hmgcoa reductase inhib(statins)	16.57	16%	2974
	beta-adrenergic blocking agents	13.48	13%	2499
	selective serotonin reuptake inhibitor (ssris)	12.02	10%	1894
	antihypertensives, angiotensin receptor antagonist	16.41	6%	1213
	calcium channel blocking agents	11.41	6%	1147

Abbreviations: OOP, out-of-pocket cost; HMG-CoA, 3-hydroxy-3-methylglutaryl co-enzyme A

© 2026 Ying R et al. *JAMA Health Forum*.

eTable 4. Associations between RTPB availability and prescription fill rates: sensitivity analyses using alternate merging criteria for medication dispense data

	Proportion of		Estimated impact of RTPB recommendations on order fill rates, percentage points, Est.				
				Average out-of-pocket cost in drug class			
	RTPB	Control	All orders	>$120.8	$50.1-$120.8	$22.6-$50.1	<$22.6
(n=38,289)							
Baseline results	55%	54%	1.2 (-1.3, 3.7)	14.7 (8.6, 20.8)	2.8 (-1.1, 6.7)	0.5 (-2.8, 3.8)	-0.5 (-3.8, 2.8)
Medication orders to fills matching criteria (Baseline: Patient MRN, Medication, Prescriber NPI, order date)							
Patient MRN, Medication, Prescriber NPI, Pharmacy ID, order date	51%	51%	0.7 (-1.7, 3.1)	11.6 (4.7, 18.5)	2.5 (-1.4, 6.4)	0.4 (-2.7, 3.5)	-1.0 (-3.9, 1.9)
Patient MRN, Medication, Pharmacy ID, order date	54%	54%	0.7 (-1.7, 3.1)	12.0 (4.7, 19.3)	2.1 (-2.0, 4.2)	1.1 (-2.0, 4.2)	-1.3 (-4.4, 1.8)
Patient MRN, Medication, order date	58%	57%	1.1 (-1.4, 3.6)	14.7 (8.4, 21.0)	2.4 (-1.7, 3.1)	1.2 (-2.1, 4.5)	-0.9 (-4.4, 2.6)
Medication order date window for orders to fills criteria (Baseline: +/- 28 days)							
+/- 1 day	51%	51%	0.8 (-1.6, 3.2)	13.7 (7.6, 19.8)	1.3 (-2.2, 4.8)	-0.3 (-3.2, 2.6)	0.1 (-3.2, 3.4)
+/- 14 day	52%	52%	0.9 (-1.5, 3.3)	13.2 (6.7, 19.7)	1.2 (-2.3, 4.7)	-0.3 (-3.2, 2.6)	0.4 (-2.9, 3.7)

Abbreviation: RTPB, real-time prescription benefits; MRN, medical record number; NPI, national prescriber identifier

[a]Given the lack of a common identifier between the datasets of prescription orders and prescription fills, orders were linked by a combination of common variables.

© 2026 Ying R et al. *JAMA Health Forum*.

eTable 5. Effects of RTPB recommendations on prescription fill rates: sensitivity analyses (1) removing conditioning on patient appearing in medication dispense data and (2) conditioning on pharmacy appearing in dispense data

	Proportion of orders filled		Estimated impact of RTPB recommendations on order fill rates, percentage points, Est. (95% CI)				
				Average out-of-pocket cost in drug class			
	RTPB	Control	All orders	>$120.8	$50.1-$120.8	$22.6-$50.1	<$22.6
No filtering (n=46,266)	33	32	0.6 (-1.4, 2.6)	6.9 (2.2, 11.6)	1 (-1.7, 3.7)	-0.7 (-3.1, 1.7)	0.7 (-2.4, 3.8)
Filter to orders for which pharmacies appear in dispense report data (n=35,688)	42	40	1.5 (-1, 4)	7.6 (2.7, 12.5)	1.7 (-1.8, 5.2)	-0.7 (-4.2, 2.8)	2.3 (-0.8, 5.4)

Note: The effect sizes in these sensitivity analyses are lower because both cases add unfilled prescriptions to the analysis. (If they had been filled, they would have been observed in our fill data and therefore included in the primary analysis.) Since fills rates in the sensitivity analyses are all lower, the percentage point difference (effect size) is smaller.

© 2026 Ying R et al. *JAMA Health Forum*.

eTable 6. Associations between RTPB availability and prescription fill rates: sensitivity analyses stratified by insurance type

	Proportion of orders filled		Estimated impact of RTPB recommendations on order fill rates, %, Adjusted est. (95% CI)				
				Average out-of-pocket cost in drug class			
	RTPB	Control	All orders	>$120.8	$50.1-$120.8	$22.6-$50.1	<$22.6
Insurance type:							
Commercial	54%	53%	1.5 (-1.2, 4.2)	15.9 (9.5, 22.2)	1.9 (-3.6, 7.4)	0.6 (-3.8, 2.6)	-0.6 (-3.8, 2.6)
Medicare	55%	55%	0.5 (-2.4, 3.4)	10.5 (1.9, 19.2)	1.7 (-27, 6.2)	0.5 (-3.5, 4.5)	-0.6 (-4.5, 3.4)

© 2026 Ying R et al. *JAMA Health Forum*.

eTable 7. Associations between RTPB availability and prescription fill rates: sensitivity analyses stratified by (a) non-mail order and (b) mail order prescriptions

(a) Excluding mail order	Proportion of orders filled		Impact of RTPB recommendations on order fill rate, percentage points	
	RTPB	Control	Unadjusted, Est. (95% CI)	Adjusted, Est. (95% CI)
All orders	57%	57%	0.8 (-2.3, 4.0)	1.7 (-0.7, 4.1)
Stratified by average out-of-pocket-cost in drug class for 30 days supply				
>$120.8	52%	35%	16.2 (8.0, 24.5)	14.5 (8.4, 20.7)
$50.1-$120.8	49%	48%	1.1 (-5.1, 7.2)	2.7 (-1.4, 6.9)
$22.6-$50.1	56%	57%	-0.9 (-4.5, 2.6)	0.7 (-2.4, 3.8)
<$22.6	62%	63%	-1.3 (-5.4, 2.9)	0.4 (-2.8, 3.6)
**(b) Mail order only**	**Proportion of orders filled**		**Impact of RTPB recommendations on order fill rate, percentage points**	
	RTPB	Control	Unadjusted, Est. (95% CI)	Adjusted, Est. (95% CI)
All orders	16%	16%	-0.1 (-5.6, 5.4)	0.3 (-3.0, 3.6)
Stratified by average out-of-pocket-cost in drug class for 30 days supply				
>$120.8	25%	9%	16.0 (1.4, 30.5)	8.2 (-3.3, 19.8)
$50.1-$120.8	18%	20%	-2.2 (-12.0, 7.6)	3.4 (-6.1, 12.9)
$22.6-$50.1	22%	20%	2.3 (-7.5, 12.1)	1.2 (-4.9, 7.2)
<$22.6	10%	11%	-1.3 (-5.4, 2.8)	-2.4 (-6.4, 1.6)

© 2026 Ying R et al. *JAMA Health Forum*.

eTable 8. Associations between RTPB availability and prescription fill rates: sensitivity analysis using two-way clustering at the practice and patient levels

	Impact of RTPB recommendations on order fill rate, percentage points	
	Unadjusted, Est. (95% CI)	Adjusted, Est. (95% CI)
All orders	0.2 (-2.7, 3.1)	1.2 (-1.3, 3.7)
Stratified by average out-of-pocket-cost in drug class for 30 days supply		
>$120.8	16.1 (8.3, 24.0)	14.5 (8.4, 20.6)
$50.1-$120.8	0.9 (-5.0, 6.8)	2.8 (-1.1, 6.7)
$22.6-$50.1	-1.6 (-5.3, 2.1)	0.5 (-2.8, 3.8)
<$22.6	-2.3 (-6.6, 2.0)	-0.6 (-3.9, 2.7)

© 2026 Ying R et al. *JAMA Health Forum*.

eTable 9. Falsification analysis: orders not eligible for RTPB recommendations

	Impact of RTPB recommendations on order fill rate, percentage points	
	Unadjusted, Est. (95% CI)	Adjusted, Est. (95% CI)
All orders	0.7 (-3.8, 5.2)	1.7 (-1.8, 5.2)
Stratified by average out-of-pocket-cost in drug class for 30 days supply		
>$120.8	4.1 (-2.2, 10.4)	2.8 (-1.5, 7.1)
$50.1-$120.8	-1.3 (-9.9, 7.3)	-0.7 (-3.4, 2.0)
$22.6-$50.1	-0.2 (-3.7, 3.3)	1.6 (-1.5, 4.7)
<$22.6	1.5 (-4.2, 7.2)	2.1 (-2.4, 6.6)

© 2026 Ying R et al. *JAMA Health Forum*.
