# Supplementary material for: Real-Time Prescription Benefit Tool Availability and Prescription Medication Fill Rates: A Post Hoc Analysis of a Cluster Randomized Clinical Trial
Source: JAMA Health Forum. 2026 Aug 14;7(8):e262739. doi: 10.1001/jamahealthforum.2026.2739 (PMC13476843; doi:10.1001/jamahealthforum.2026.2739)
Supplement: Supplementary file 3 [file jamahealthforum-e262739-s003.pdf]

Data Sharing Statement

Ying. Real-Time Prescription Benefit Tool Availability and Prescription Medication Fill Rates. *JAMA Health Forum*. Published August 14, 2026. doi:10.1001/jamahealthforum.2026.2739

Data
Additional Information: ClinicalTrials.gov number, NCT04940988; American Economic Association Registry number AEARCTR-0006909
Data available: Yes
Data types: Data dictionary
How to access data: Data will be available upon request.
When available: With publication

Supporting Documents
Document types: None

Additional Information
Who can access the data: Researchers requesting the data
Types of analyses: For research
Mechanisms of data availability: After approval of a proposal
